# HOW POLITICO-ECONOMIC SYSTEMS CAN SHAPE THE ATTITUDE TOWARD ELDERLY CARE: LESSONS FROM THE GERMAN REUNIFICATION

**DOI:** 10.1093/geroni/igz038.2496

**Published:** 2019-11-08

**Authors:** Freya Diederich, Hans-Helmut König, Christian Brettschneider

**Affiliations:** University Medical Center Hamburg-Eppendorf, Hamburg, Germany

## Abstract

Migration flows have been rising over the past decades and are not expected to mitigate in the future. Migrants brings along values and preferences that were shaped by their origin countries, among those, their perceptions of how societies should care for the elderly. In this study, we examine how the attitude towards informal care is shaped by the politico-economic system an individual grew up in and if this attitude adjusts over time once an individual lives in a different system. After the fall of the Berlin Wall, Eastern Germans were exposed to the market-oriented western economy, a natural experiment that allows us to address these issues. By analyzing data from the German Family Panel (2009/10-2015/16), we assess differences in attitudes towards informal care among four birth cohorts that were born during the German separation (N=11,966) using random-effects models. We control for socio-demographic factors as well as the institutional and economic environment an individual lives in. The results reveal that, on average, older generations that grew up in East Germany exhibit up to a 6.7% (95% CI, 0.03-0.1) higher willingness to provide informal care to their parents than older generations that grew up in West Germany. There is no significant difference in younger birth cohorts. Attitudes do not significantly converge over the observed time horizon. The results provide evidence that individuals’ attitude towards informal care is deeply shaped by the system they grew up in such that migration flows can influence the supply of informal care in the future.

